# Estimation Model of Potassium Content in Cotton Leaves Based on Wavelet Decomposition Spectra and Image Combination Features

**DOI:** 10.3389/fpls.2022.920532

**Published:** 2022-07-13

**Authors:** Qiushuang Yao, Ze Zhang, Xin Lv, Xiangyu Chen, Lulu Ma, Cong Sun

**Affiliations:** The Key Laboratory of Oasis Eco-Agriculture, College of Agriculture, Shihezi University, Shihezi, China

**Keywords:** hyperspectral imaging, potassium content in leaves, continuous wavelet transform, gray level co-occurrence matrix, cotton, growth stage

## Abstract

Potassium (K) is one of the most important elements influencing cotton metabolism, quality, and yield. Due to the characteristics of strong fluidity and fast redistribution of the K in plants, it leads to rapid transformation of the K lack or abundance in plant leaves; therefore, rapid and accurate estimation of potassium content in leaves (LKC, %) is a necessary prerequisite to solve the regulation of plant potassium. In this study, we concentrated on the LKC of cotton in different growth stages, an estimation model based on the combined characteristics of wavelet decomposition spectra and image was proposed, and discussed the potential of different combined features in accurate estimation of the LKC. We collected hyperspectral imaging data of 60 main-stem leaves at the budding, flowering, and boll setting stages of cotton, respectively. The original spectrum (R) is decomposed by continuous wavelet transform (CWT). The competitive adaptive reweighted sampling (CARS) and random frog (RF) algorithms combined with partial least squares regression (PLSR) model were used to determine the optimal decomposition scale and characteristic wavelengths at three growth stages. Based on the best “CWT spectra” model, the grayscale image databases were constructed, and the image features were extracted by using color moment and gray level co-occurrence matrix (GLCM). The results showed that the best decomposition scales of the three growth stages were CWT-1, 3, and 9. The best growth stage for estimating LKC in cotton was the boll setting stage, with the feature combination of “CWT-9 spectra + texture,” and its determination coefficients (*R*^2^val) and root mean squared error (RMSEval) values were 0.90 and 0.20. Compared with the single R model (*R*^2^val = 0.66, RMSEval = 0.34), the *R*^2^val increased by 0.24. Different from our hypothesis, the combined feature based on “CWT spectra + color + texture” cannot significantly improve the estimation accuracy of the model, it means that the performance of the estimation model established with more feature information is not correspondingly better. Moreover, the texture features contributed more to the improvement of model performance than color features did. These results provide a reference for rapid and non-destructive monitoring of the LKC in cotton.

## Introduction

Potassium (K) is an essential and favorite nutrient element in the growth of cotton. The level of K directly affects the growth and development of cotton and the quality of fiber (Pettigrew, 2008; Lewis et al., 2021). Affected by the parent material of soil formation, the soil in Xinjiang, China is rich in K. The content of available K in arable soil tends to be high in the north and low in the south, but in recent years, cotton fields in some areas have been deficient in K (Tian et al., 2020; Wang et al., 2021). Furthermore, Xinjiang has high-quality and high-yield cotton and a large demand for soil nutrients. Therefore, an excessive supply of potash fertilizer is usually used to avoid production reduction due to lack of the K, resulting in an increase in cotton production costs. However, the accurate method of element determination is time-consuming and laborious, so it is of great significance to monitor the potassium content in cotton leaves (LKC) quickly and without damage for the healthy growth of cotton, the recommendation of fertilizer application amounts and the reduction in resource waste.

Proximal hyperspectral remote sensing technology has become an effective means to evaluate precision agriculture (Pandey et al., 2017; Li et al., 2019), which can be divided into imaging spectra and non-imaging spectra. They can collect hyperspectral reflectance data from the visible, near-infrared (NIR) and short-wave infrared (SWIR) regions of the electromagnetic spectrum (Mertens et al., 2021), so that a wide variety of physiological traits of crops can be studied, such as crop nutrient deficiency (Furlanetto et al., 2021; Jiang et al., 2021; Mahajan et al., 2021, photosynthetic efficiency (El-Hendawy et al., 2017), water stress (Sun et al., 2021; Zhou et al., 2021), chlorophyll fluorescence (Zhao et al., 2021), heavy metal pollution (Lin et al., 2021) and early plant disease detection (El-Hendawy et al., 2017; Barros et al., 2020). On the other hand, hyperspectral imaging can simultaneously obtain the target spectrum and image information, and is regarded as a technique with high-throughput plant phenotype potential (Pandey et al., 2017). Although there are many studies on nutrition monitoring using near-end hyperspectral imaging, most of them focus on quantitative monitoring and diagnosis of crop nitrogen (N), such as wheat (Mahajan et al., 2014; Jiang et al., 2021), rice (Men et al., 2021), maize (Furlanetto et al., 2021), cotton (Oliveira et al., 2020), rape (Liu et al., 2020a), soybean (Chen et al., 2019), orange (Osco et al., 2019, 2020a), tea (Wang et al., 2020) and mango (Mahajan et al., 2021). At present, the quantitative monitoring research on crop K is also gradually carried out, but more studies often analyze the K together with other elements (Liu et al., 2020b; Osco et al., 2020a,b; Mahajan et al., 2021), and there are few studies only on the characteristics of single the K nutrient element. Indeed, a large group of K^+^ transporters and channels has been identified in plants (Gierth and Maser, 2007), and cytoplasmic concentration of K^+^ is maintained around 80–150 MM (Ahmad and Maathuis, 2014). Preserving this concentration range is important for many physiological processes as the enzyme activations, and stabilization of protein synthesis (Villette et al., 2020). These processes are present in all tissues and subcellular compartments of cells, which enables the precise quantification of foliar K attributes of the foliage. It has been shown that the 550–700 and 1,390–1,880-nanometer (nm) wavelengths were the best wavelengths to explain the difference in nutrient levels of N, P, and K in cotton (Oliveira et al., 2020; Wang et al., 2020). Thus, the research utilizing sensitive characteristic wavelengths or vegetation indexes to identify and estimate the K deficiency are common method in rice (Das et al., 2020), wheat (Hussain et al., 2017), and maize (Furlanetto et al., 2021). However, to which extent the K can be estimated using hyperspectral requires further investigations.

Continuous wavelet transform (CWT) has attracted increasing attention in image and spectral signal decomposition due to its rich wavelet basis function, multi-resolution, and time-frequency locality (Chen et al., 2010; Yue et al., 2020). Because the CWT can perform multi-scale decomposition of spectrum and has good performance in characteristic wavelength selection and fine spectral signal extraction (Chen et al., 2019), it has been widely used in crop biochemical parameter inversion of hyperspectral data (Zhang et al., 2014), including estimating the above-ground biomass of wheat (Yao et al., 2018; Yue et al., 2020), analyzing the relationship between leaf copper content and spectrum (Lin et al., 2021) and rapidly detecting the chlorophyll fluorescence parameters of potato leaves (Zhao et al., 2021). Therefore, it is of great significance to improve the accuracy of spectral monitoring to construct a quantitative regression relationship between the wavelet coefficients and nutrient parameters (Mahajan et al., 2014).

As an imaging spectrometer can provide very high spatial and spectral resolution data (Pandey et al., 2017), it is necessary to consider the spatial information (e.g., color and texture) in hyperspectral images in addition to the spectral information to estimate crop nutrients. Image color can express the color distribution and range of image, while image texture reflects the information of uniformity, sharpness and spatial arrangement of image gray distribution. Although there are few studies on the role of image features in hyperspectral nutrient monitoring, it has important application potential in the field of hyperspectral imaging (Jiang et al., 2021). Zheng et al. (2017) extracted 14 vegetation indices related to color features to segment corn, and the accuracy rate over 90.19%. Zou et al. (2019) segmented broccoli seedlings from weeds and soil by extracting GLCM features and color features, and achieved higher accuracy. In the existing studies have demonstrated that the K deficiency causes discoloration of crop leaf tips and edges [such as wheat (Mahajan et al., 2014), rice (Sun et al., 2018), soybean (Ghosal et al., 2018), and cotton (Oliveira et al., 2020)], then gradually spread to the center of the leaf, develop into brown spots, and finally wither and necrosis, resulting in changes in leaf color and texture (Laddi et al., 2013). Also, vegetation coverage and NDVI value are significantly reduced (Severtson et al., 2016). Besides, through the calculation of crop RGB image, it was found that the extension rate of the K deficient leaves slowed down and the wilting rate accelerated (Sun et al., 2018). However, the potential for the image features of leaf hyperspectral imaging data for estimating crop nutrients stress (e.g., K) is not well documented.

Hence, using the high-resolution proximal hyperspectral imaging data of cotton leaves in different growth stages, this study proposed an estimation model of the LKC in cotton based on the combined characteristics of “CWT spectra + image.” The main objectives of this study were to (1) clarify the characteristics of hyperspectral response of cotton LKC at different growth stages, and the effective characteristic wavelengths of the best decomposition scale was determined combined with CWT and PLSR, (2) construct a gray image database of characteristic wavelengths in different growth periods to extract and screen sensitive image features, and (3) evaluate the potential of different “CWT spectra + image” combination features to estimate the LKC of cotton at different growth stages.

## Materials and Methods

### Experimental Design

The research area was located in Erlian (85°59′41″E, 44°19′54″N), the teaching experiment field of Shihezi University. Sunshine duration is 2,721–2,818 h, ≥ 0°C active accumulated temperature is 4,023–4,118°C, ≥10°C active accumulated temperature is 3,570–3,729°C and frost-free period is 168–171 days. The soil texture was loam, and the 0–20 cm soil layer contained 19.06 g·kg^−1^ organic matter, 12.8 mg·kg^−1^ total nitrogen, 20.8 mg·kg^−1^ available phosphorus, and 165.1 mg·kg^−1^ available potassium. The soil pH is 8.17 and electrical conductivity (EC) is 0.42 ms·cm^−1^. During the whole growth period of cotton, nitrogen, phosphorus, and potassium fertilizer were applied with water drops. The urea (N, 46%) of 276 kg·hm^−2^, monoammonium phosphate (P_2_O_5_, 61%) of 174 kg·hm^−2^ and potassium sulfate (K_2_O, 50%) was used as a potassium fertilizer. A total of 9-times drips were given during the whole growth period, and the fertilization ratios of the three fertilizers (N, P, and K) were 2.5, 7.7, and 0% (June 7), 7.5, 11.7, and 6.7% (June 15), 7.5, 11.7, and 6.7% (June 24), 12.5, 19.2, and 20% (July 2), 20, 19.2, and 20% (July 18), 25, 15.4, and 13.3% (July 26), 15, 15.4, and 13.3% (Aug 5), 10, 0, and 13.3% (Aug 15), 0, 0, and 6.7% (Aug 25). At each fertilization, the three fertilizers weighed in proportion are poured into the corresponding differential pressure fertilization tank to dissolve, and then drip irrigation was applied to the plot.

**Figure 1:**
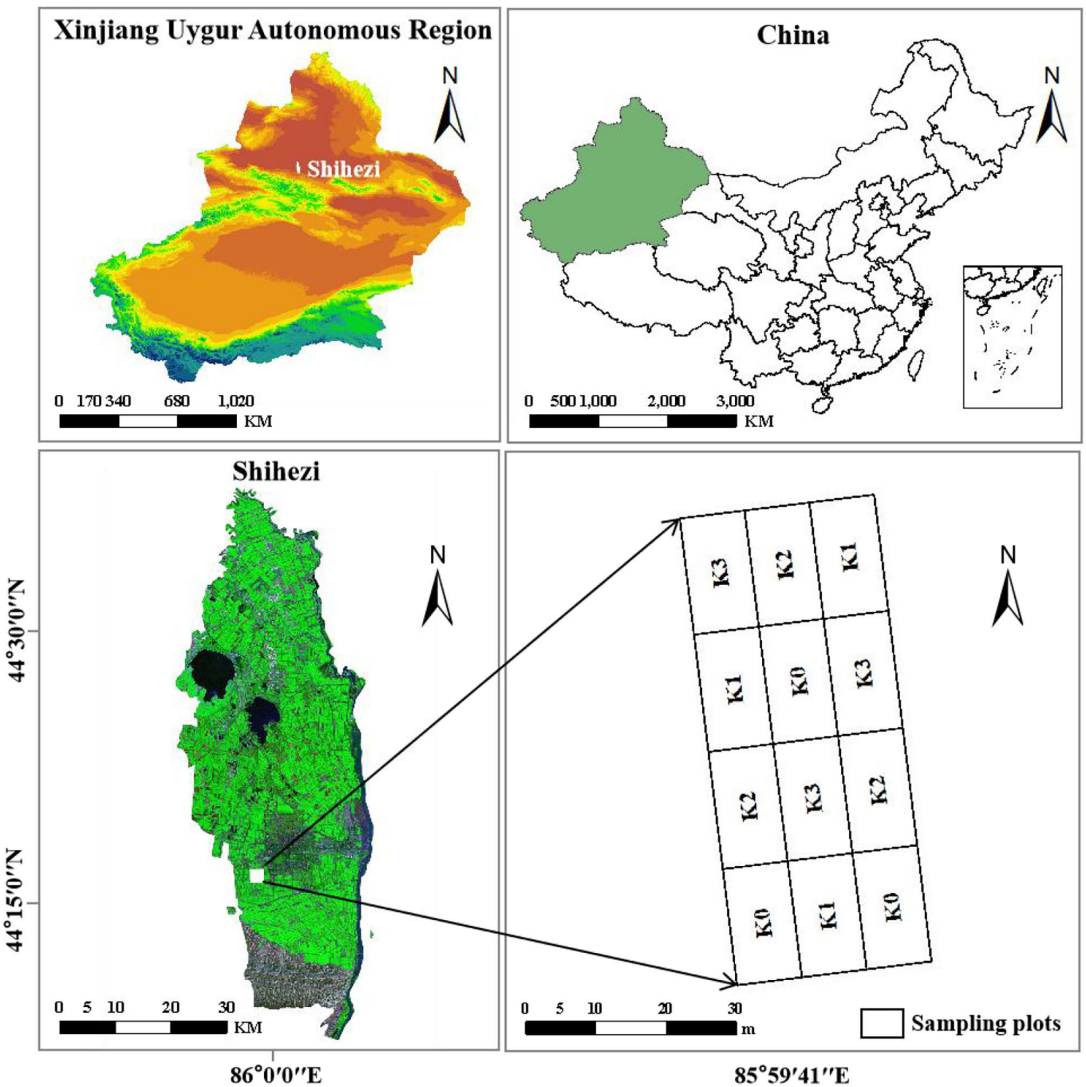
The study site and the location of the experiments.

The experiment was carried out in the study area from April to September 2020. The variety Xinluzao 53 was selected for the experiment. The planting pattern was “one film, three tubes, and six rows” and the plant spacing was 10 + 66 + 10 cm. Four K application levels were set, namely, blank (0 kg·hm^−2^), low K (75 kg·hm^−2^), conventional K (225 kg·hm^−2^), and high K (375 kg·hm^−2^). We used a random block design with 3 replicates on a total of 12 plots with a single plot area of 25 m^2^ (Figure 1). The sowing date was 18 April 18 2020, the topping date was 9 July 202 and the sampling periods were the budding stage (30 June 2020), flowering stage (12 July 2020) and boll setting stage (30 July 2020). Five pieces of cotton main-stem leaves with similar growth in the middle and upper parts were randomly collected from each plot, a total of 60 main-stem leaves were collected in one growth period, and a total of 180 main-stem leaves were collected in three growth periods.

### Hyperspectral Image Data Acquisition

**Figure 2:**
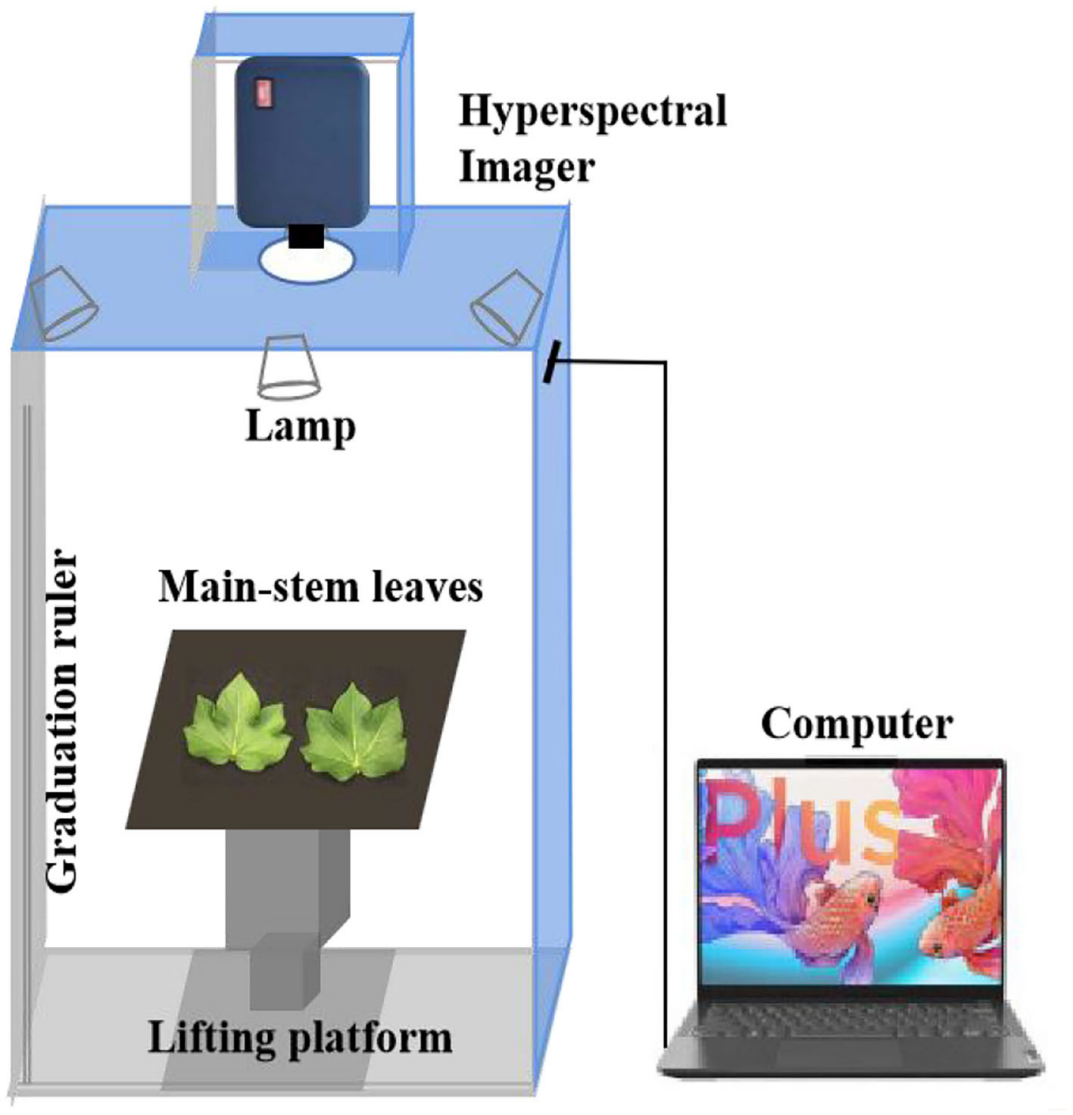
Hyperspectral image acquisition system. System consisting of a hyperspectral imager, dark box, lighting system, lifting platform, and a computer.

The SOC710-VP portable visible-near-infrared hyperspectral imaging spectrometer (Surface Optics Corporation, USA) was used for data acquisition. The spectral resolution is 5 nm, the image resolution is 692 × 520 and the spectral range is 376–1,044 nm, with a total of 128 bands. After removing the front and rear spectral noises, each hyperspectral image cube selected a wavelength in the range of 400–950 nm, with a total of 106 image bands. To reduce the influence of natural light, all images were captured in a dark box (Figure 2).

Cotton main-stem leaves are the main source organs providing assimilates to cotton bolls (Pace et al., 1999). Fresh main-stem leaves at the three key growth stages were selected to be tiled in a dark box with a low-reflectivity black background plate according to the order of leaf position. At the same time, a standard gray plate was placed 5 cm away from the leaf edge to assist black-and-white correction. To reduce the influence of light source intensity, exposure time, and dark current in the sensor during spectral scanning, the instrument should be preheated for 30 min. The scanning parameters of the hyperspectral imager were as follows: object distance, 88 cm, scanning rate, 150–200 frames·s^−1^, aperture, 5.6. The collected spectral data were digital (DN) and were converted into spectral reflectance through spectral calibration and radiometric calibration in SRAnal 710 software according to the grayscale reference panel in each original image. The average spectral reflectance of the whole leaf was extracted as the original spectral data of this sample (Lin et al., 2021).

### Determination of Total Potassium Content in Plants

The total potassium content in leaves was determined using a H_2_SO_4_-H_2_O_2_ flame photometer (Bao, 2000). Fresh leaves were dried at 85°C for 30 min and then at 105°C until reaching a constant weight. The dried leaf samples were ground, weighed and then digested with H_2_O_2_-H_2_SO_4_, and the K was determined using a laboratory flame photometer (FP640, Yidian Co., Ltd, Shanghai, China). The total LKC was calculated according to the following formula:


(1) $$\begin{array}{l} K\left(\%\right)=\frac{\rho \times V\times ts\times 10^{-4}}{m} \end{array}$$

where ρ is the mass concentration of K obtained from the standard curve (ug·mL^−1^), *V* is the measuring liquid volume (ml), *t*_*s*_ is the separation multiple, and *m* is the dry sample mass (g).

### Data Processing

#### Continuous Wavelet Transform

Continuous wavelet transform (CWT) is an effective signal processing tool to decompose an original signal into multidimensional signals, mainly including discrete wavelet transform and continuous wavelet transform (Liu et al., 2020b). In the CWT, the algorithm uses the selected mother wavelet to decompose the hyperspectral data into a series of wavelet coefficients of different scales, which is a linear transformation. Its transformation formula is as follows:


(2) $$\begin{array}{l} W_{f}\left(a,b\right)=\int_{-\infty }^{\infty }f\left(\lambda \right)\psi_{a,b}d\lambda \end{array}$$

(3) $$\begin{array}{l} \psi_{a,b}\left(\lambda \right)=\frac{1}{\sqrt{a}}\psi \left(\frac{\lambda -b}{a}\right) \end{array}$$

where *f* (λ) is the leaf hyperspectral reflectance, λ is the wavelength within 400–950 nm, ψ_*a,b*_ is the wavelet basis function, a is the scale factor, *b* is the translocation factor, and *W*_*f*_(*a, b*) is a two-dimensional matrix, including *i* and *j*, where *i* represents the decomposition scale (*i* = 1, 2, 3, …, *m*) and *j* represents the band range of the spectrum (*j* = 1, 2, 3, …, *n*), forming an *m* by *n* matrix. In this study, the spectral data of cotton leaves at three growth stages were obtained, and each leaf sample included 106 bands. As the setting of the decomposition scale has a certain influence on spectral feature recognition (Liu et al., 2020; Lin et al., 2021), the decomposition scale in this study was set as 2^1^, 2^2^, 2^3^, …, 2^10^, scales 1–10. Among them, the decomposition scales 1–3 and 4–7 belong to low frequency and middle frequency, respectively, and the rest belong to high frequency. Sym2 was selected as the wavelet basis function, and then, a PLSR model was used to quantitatively analyze the relationship between the wavelet coefficients of each decomposition scale and the LKC so as to determine the optimal decomposition scale and effective wavelength.

#### Selection Method of Characteristic Variables

Different feature selection methods lead to different features being selected. To select spectral and image features sensitive and stable to the LKC in cotton, the competitive adaptive reweighted sampling (CARS), and random frog (RF) algorithms were used in this study to screen features.

Competitive adaptive reweighted sampling selects wavelength points with a large coefficient absolute value in the model through the Monte Carlo strategy and removes wavelength points with a low weight (Sun et al., 2021). The subset with the lowest root mean squared error of cross validation (RMSECV) value is retained as the feature selection result by cross-validation. In this study, the Monte Carlo strategy was set to run 50 times, using 5-fold cross-validation.

Random frog is an algorithm to measure the importance of variables (El-Hendawy et al., 2017). The main steps are as follows: (1) a subset of initial variables containing m variables is randomly initialized, (2) variables in the initial variable subset are continuously selected into the candidate subset, and the number of variables in the candidate subset increases and decreases with the number of iterations, (3) the selection probability of each variable is calculated as a measure of the importance of the variable, and (4) the characteristic wavelength is selected according to the probability of the occurrence of recorded variables in each iteration. In this study, the selection probability of each wavelength was used to screen the feature information, and the running results are presented in descending order. The number of iterations was set as 10,000, and the selection probability thresholds of the three growth periods were 0.40, 0.21, and 0.23, respectively.

#### Image Feature Extraction

The most common gray level co-occurrence matrix (GLCM) algorithm was adopted to extract texture features (Yang et al., 2021). In this study, the energy (ENE), entropy (ENT), contrast (CON), correlation (COR), and their mean (MEA) and variance (VAR) in four directions were calculated by using the gray comatrix function. The calculation equation is shown in Table 1, where the *P*(*i, j*) is the value of the GLCM in the *i*th row and *j*th column, *k* is the number of gray levels in the GLCM. The gray level is 256, the step size is 1, the angle is 0°, 45°, 90°, and 135°. Finally, each characteristic wavelength grayscale image will eventually produce 24 (4 × 4 + 8) texture features.

**Table 1:** The calculation equations for the characteristics of the GLCM.

**Feature**	**Equation**
ENE	$ENE=\overset{k}{\underset{i=0}{\sum }}\overset{k}{\underset{j=0}{\sum }}P{\left(i,j\right)}^{2}$
ENT	$ENT=-\overset{k}{\underset{i=0}{\sum }}\overset{k}{\underset{j=0}{\sum }}P\left(i,j\right)\cdot \ln\;P\left(i.j\right)$
CON	$CON=\overset{k}{\underset{i=0}{\sum }}\overset{k}{\underset{j=0}{\sum }}P\left(i,j\right)\cdot {\left(i,j\right)}^{2}$
COR	$COR=\overset{k}{\underset{i=0}{\sum }}\overset{k}{\underset{j=0}{\sum }}P\left(i,j\right)\frac{\left(i-MEA\right)\cdot \left(j-MEA\right)}{\sqrt{VARi\cdot VARj}}$
MEA	$MEA=\overset{k}{\underset{i=0}{\sum }}\overset{k}{\underset{j=0}{\sum }}P\left(i,j\right)\cdot i$
VAR	$VAR=\overset{k}{\underset{i=0}{\sum }}\overset{k}{\underset{j=0}{\sum }}P\left(i,j\right)\cdot {\left(i-MEA\right)}^{2}$

Color moments are used to represent the color distribution in the image (Ge et al., 2021). Since the color information is mainly distributed in low-order moments, first-order moments (mean, MEA), second-order moments (variance, VAR), and third-order moments (skewness, SKE) are sufficient to express the color distribution of the image. Its formula is as follows:


(4) $$\begin{array}{l} MEA=\overset{n}{\underset{j=1}{\sum }}\frac{1}{n}Pij \end{array}$$

(5) $$\begin{array}{l} VAR=\sqrt{\frac{1}{n}\sum_{j=1}^{n}(Pij-MEAi{)}^{2}} \end{array}$$

(6) $$\begin{array}{l} SKE=\sqrt[{3}]{(\frac{1}{n}\sum_{j=1}^{n}P{\left(Pij-MEAi\right)}^{3}} \end{array}$$

where *Pij* is the color value of the *j*th pixel on the *i*th color channel, *i* is the number of color channels of the image. The image in this study is grayscale image, so *i* = 1; MEAi is the color mean of the *i*th color channel of all pixels. Finally, each characteristic wavelength grayscale image will eventually produce three color features.

Notably, the named representation of combined features is as follows: (1) The characteristic wavelength-texture feature-direction, such as 400 nm-ENE-0°, which means the texture feature is ENE in the 0° direction of the 400-nm grayscale image and (2) characteristic wavelength-color feature, such as 400-nm MEA, which means the color feature is MEA of the 400-nm grayscale image.

### Modeling and Analysis Methods

Partial least squares regression (PLSR) is one of the most widely used modeling methods in spectral analysis, which can be used for dimensionality reduction and comprehensive screening of spectral data, with high modeling stability and reliability. The PLSR is widely favored in hyperspectral analysis (Lin et al., 2021; Zhao et al., 2021) because it can solve the collinearity and overfitting characteristics of hyperspectral data compared with other multivariate models.

The determination coefficients (*R*^2^) and RMSE values were used to evaluate the performance of the model. In general, better performing models have higher *R*^2^ and lower RMSE values. Original hyperspectral data were extracted by ENVI5 3. The CWT, PLSR, and GLCM analyses of leaf spectral data were carried out by Matlab R2018a (The MathWorks, Inc., Natick MA, USA). Origin 2020 was used for creating graphs (OriginLab Corporation, Northampton, MA, USA).


(7) $$\begin{array}{l} {\text{R}}^{2}=1-\frac{\overset{n}{\underset{i=1}{\sum }}{\left(y_{i}-{ŷ}_{i}\right)}^{2}}{\overset{n}{\underset{i=1}{\sum }}{\left(y_{i}-\bar{y}\right)}^{2}} \end{array}$$

(8) $$\begin{array}{l} \text{RMSE}=\sqrt{\frac{1}{n}\overset{n}{\underset{i=1}{\sum }}{\left(y_{i}-{ŷ}_{i}\right)}^{2}} \end{array}$$

where *R*^2^cal is expressed as the determination coefficient of calibration sets, *R*^2^val is expressed as the determination coefficient of validation sets, *n* is the number of samples, *y*_*i*_ and ŷ_*i*_, are, respectively, the measured and estimated values of sample *i* in the corresponding sample set, ȳ is the average value of *y*_*i*_.

## Results

### Analysis of Spectral Characteristics

#### Statistical Data of Cotton Leaf Sample Set

The total LKC in three key growth stages of cotton was measured (Table 2). The concentration gradient method (Liu et al., 2015) was used to divide the total samples into 40 calibration sets and 20 validation sets in a ratio of 2:1. The range of the K content in the calibration set including the validation set was 3.56–0.51, 2.80–0.57, and 2.30–0.40% in the three growth stages, respectively, indicating that the calibration set could well represent the entire datasets. The coefficients of variation in the calibration sets and validation sets were both between 37 and 50%, show that the LKC in cotton studied had a wide range and had good representativeness and coverage.

**Table 2:** Statistical results of calibration and validation sets.

**Growth Stage**	**Sets**	**Size**	**Max. (%)**	**Min. (%)**	**Mean (%)**	**SD^**a**^ (%)**	**CV^**b**^ (%)**
Budding stage	All sets	60	3.56	0.51	1.63	0.81	49.47
	Calibration sets	40	3.56	0.51	1.63	0.81	49.78
	Validation sets	20	3.51	0.51	1.64	0.82	50.12
Flowering stage	All sets	60	2.80	0.57	1.18	0.44	37.47
	Calibration sets	40	2.80	0.57	1.18	0.45	38.20
	Validation sets	20	2.43	0.57	1.18	0.43	36.93
Boll setting stage	All sets	60	2.30	0.40	1.25	0.56	44.67
	Calibration sets	40	2.30	0.40	1.25	0.56	44.88
	Validation sets	20	2.30	0.46	1.25	0.57	45.42

a *SD, standard deviation*.

b *CV (%), coefficient of variation*.

#### Spectral Reflectance Analysis

**Figure 3:**
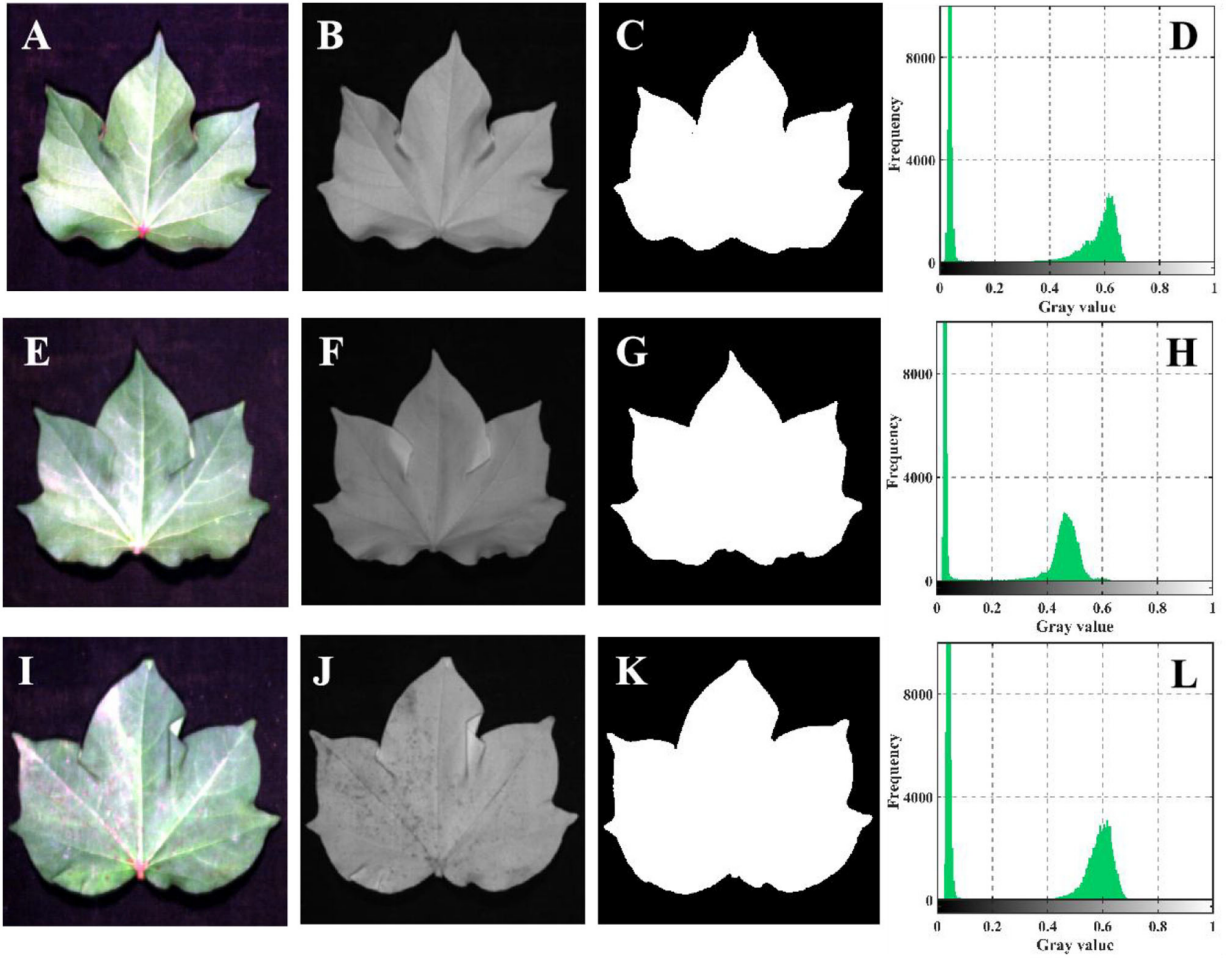
ROI extraction process by single band threshold segmentation. **(A–D)** Budding stage. **(E–H)** Flowering stage. **(I–L)** Boll setting stage. **(A,E,I)** The true color picture synthesized by ENVI cannot completely represent the true color of leaf. **(B,F,J)** The grayscale image of 800 nm. This grayscale image can display the outline of cotton leaves more completely in terms of brightness and clarity, so 800 nm is selected as the segmentation band. **(C,G,K)** Mask image. **(D,H,L)** Gray histogram.

The single-band threshold segmentation method (Figure 3) was used to extract the average spectrum of the whole cotton leaves from hyperspectral images as the original spectrum (*R*) and a region of interest (ROI). The threshold was set to 0.25–0.3098, and the sample area lower than 0.25 was the background plate.

**Figure 4:**
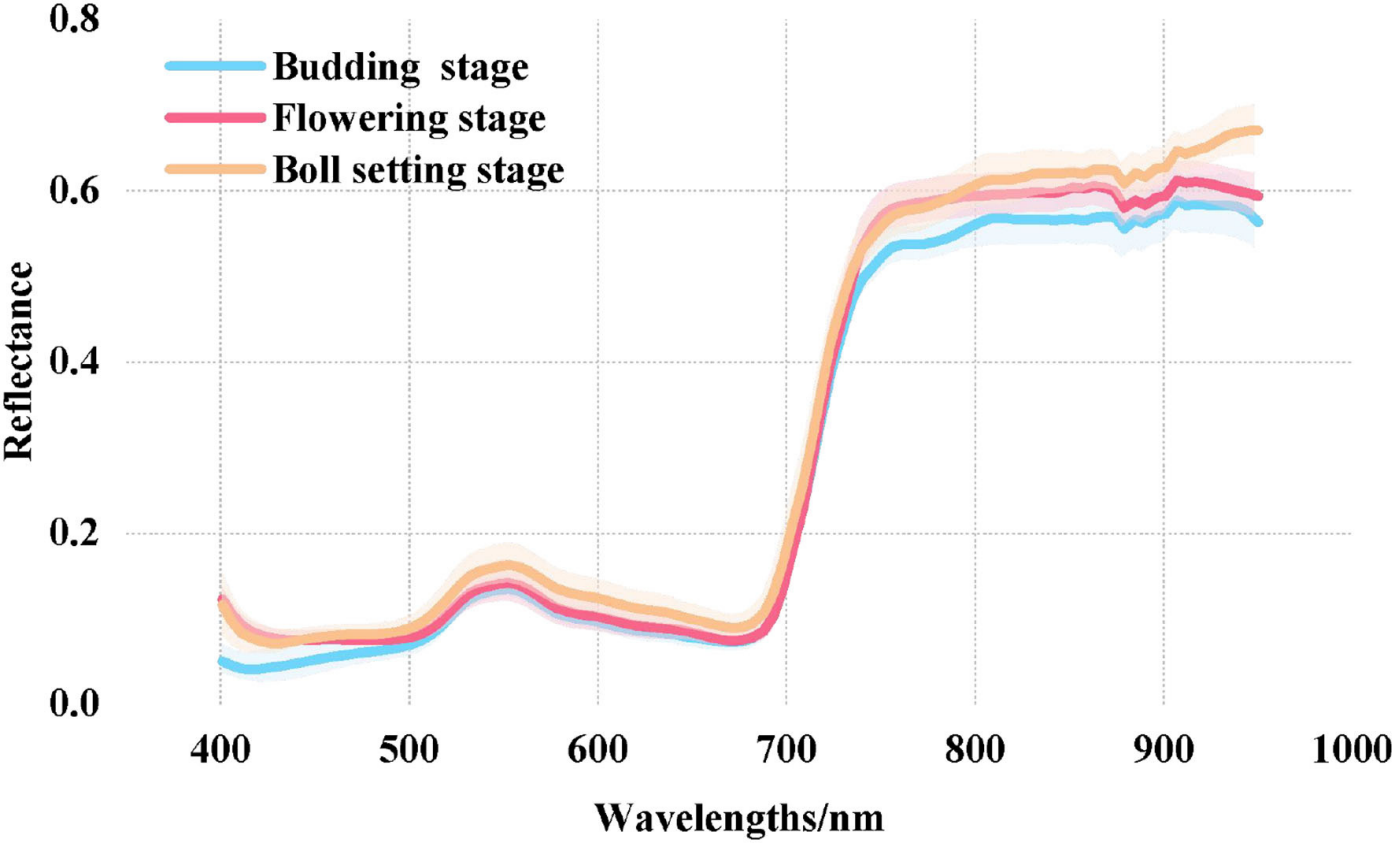
R spectral curves of the LKC in cotton at different growth stages.

The spectral curves of cotton leaves at the three growth stages were consistent with the spectral characteristics of green plants (Figure 4). There were strong absorption peaks at 450 and 680 nm and a strong reflection peak at 550 nm. Due to the many cavities in the mesophyll sponge structure, the reflectance increases sharply near the red edge region (690–760 nm), and a highly reflective platform appears in the NIR region (760–950 nm). Among them, the spectral reflectance of cotton leaves at different growth stages differed significantly in the NIR region, which showed as boll setting stage > flowering stage > budding stage. This may be because after mid-July, the redistribution and utilization of the K nutrients during fruit development and the fluidity of potassium make the K in leaves gradually transfer to cotton bolls at the flowering and boll setting stages (Singh et al., 2019), leading to the decrease in LKC, while the lack of the K in leaves increases the thickness of leaves, and the palisade tissue and parenchymal cells shrink and partially break (Zhao et al., 2001; Ramírez-Soler et al., 2021). Finally, the spectral reflectance of the boll setting stage was higher than that of the budding stage in the NIR region.

#### Correlation Analysis Between CWT Spectra and the LKC

**Figure 5:**
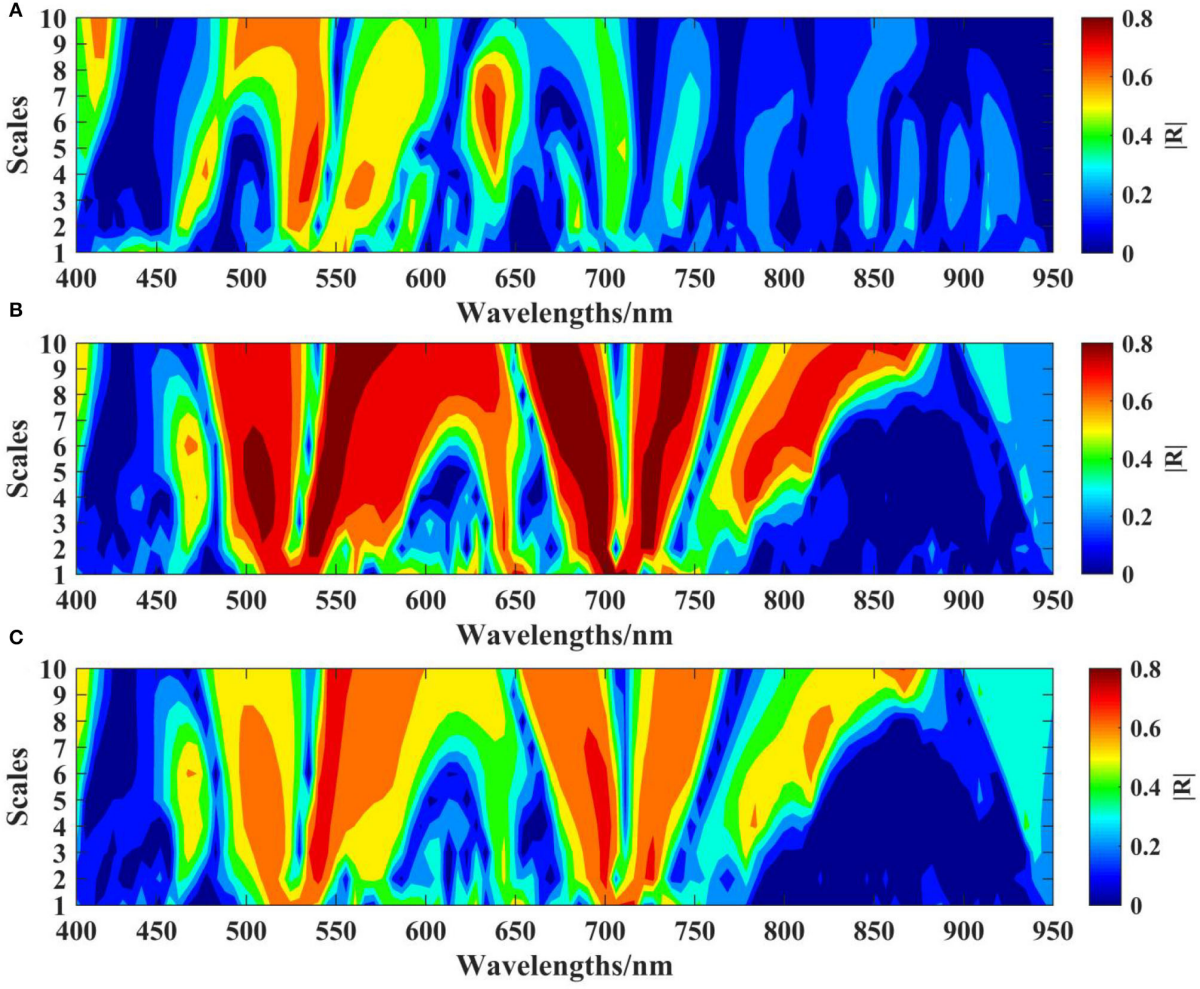
Correlation analysis between the LKC and CWT coefficient. **(A)** Budding stage. **(B)** Flowering stage. **(C)** Boll setting stage.

The R spectrum of cotton leaves was decomposed by the CWT at 10 scales. Correlation analysis was performed between the wavelet coefficients generated under each decomposition scale and the LKC, and the results were expressed as the absolute value of the correlation coefficient (|*R*|). The correlation between the wavelet coefficient and the LKC was relatively high, especially at the flowering stage of cotton growth (Figure 5). Under the calculation of different scales and movement factors, the regions with high correlation at the budding stage are mainly focused in the range of 500–550 and 640–660 nm on the mesoscales 3–5 and 7, with the highest correlation |*R*| = 0.74 (Figure 5A). The flowering stage showed obvious regional distribution, with a high correlation in the low dimension on scale 3, with the highest correlation |*R*| = 0.86 (Figure 5B). At the boll setting stage, the high-correlation area was mainly distributed in mesoscales 3–6, and the highest correlation |*R*| = 0.70 (Figure 5C). In addition, the wavelengths of 500–530, 640–660, and 740–760 nm showed higher correlation in the three growth stages.

#### Characteristic Wavelength Screening of R Spectrum and CWT Spectra

**Figure 6:**
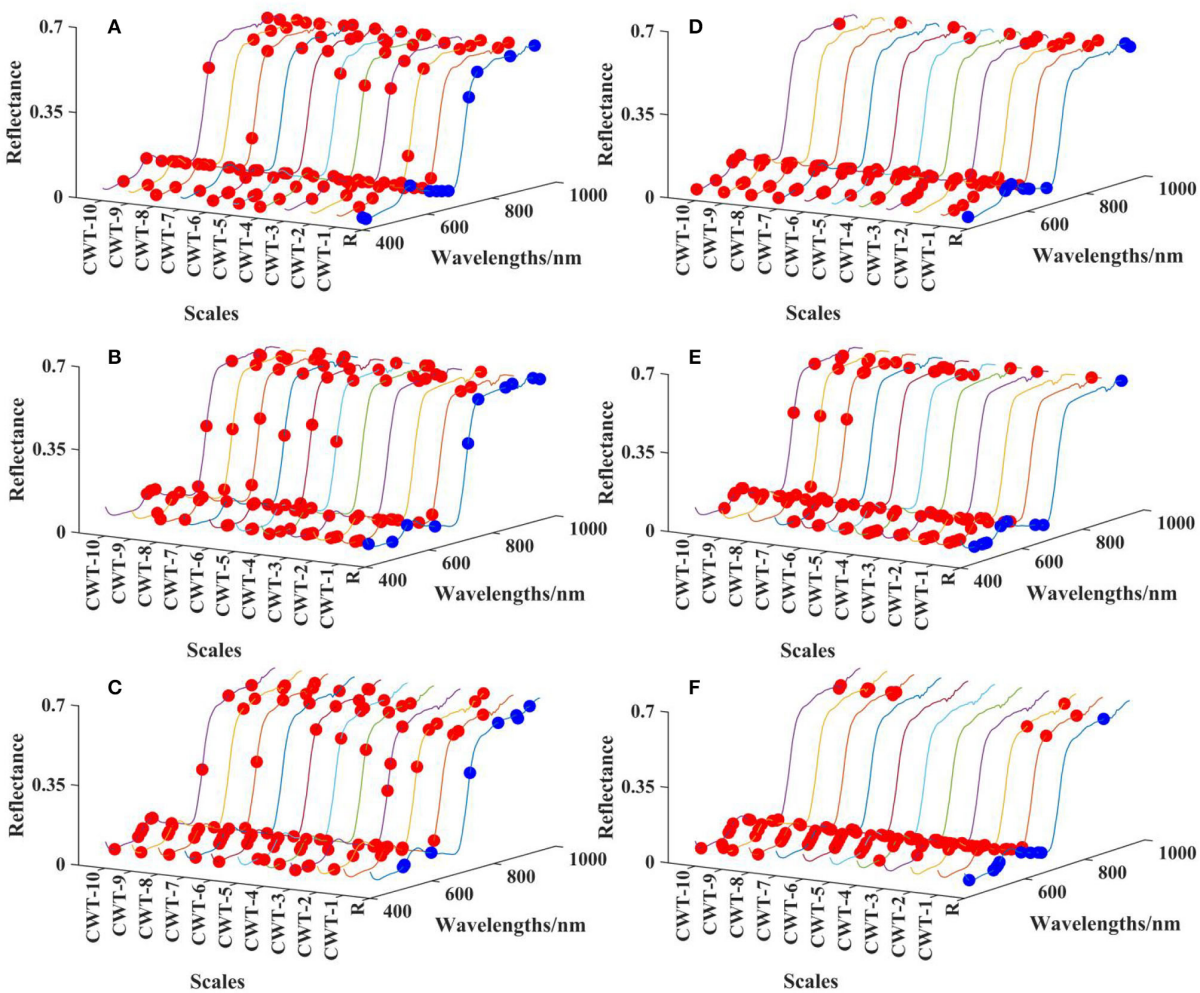
Characteristic wavelengths of the R spectrum and CWT spectra screened by CARS and RF algorithms. The red and blue dots represent the characteristic wavelengths of CWT spectra and R spectrum, respectively. **(A)** Budding stage, CARS. **(B)** Flowering stage, CARS. **(C)** Boll setting stage, CARS. **(D)** Budding stage, RF. **(E)** Flowering stage, RF. **(F)** Boll setting stage, RF.

To further screen out characteristic wavelengths for the rapid estimation of the LKC in cotton, reduce the analytical dimension of spectral data and highlight the timeliness and convenience of spectral monitoring, the CARS and RF algorithms were selected to screen the R spectra and the CWT spectra (scales 1–10) of the three growth periods, and the selected characteristic wavelengths did not exceed 10. In the whole spectrum, the selected characteristic wavelengths of the three growth stages were similar, but there are significant differences in the screening methods of different characteristic wavelengths. The characteristic wavelengths selected based on the CARS algorithm were evenly distributed in the range of 400–950 nm, mainly located in the visible (500 nm), red edge (700 nm), and NIR regions (900 nm) (Figures 6A–C). The characteristic wavelengths screened by the RF algorithm were mainly concentrated in the visible and NIR regions in the whole spectrum, but more characteristic wavelengths appear in the visible region (Figures 6D–F). The characteristic wavelengths located in the visible region reflect the information of leaf pigment, especially the characteristic bands distributed near the strong absorption and reflection of chlorophyll. Red edge is closely related to the physical and chemical parameters of plants, is generally used to describe the health status of plants and is affected by leaf pigment and leaf area index. The characteristic wavelengths were in the range of 800–950 nm, reflecting the structure of cotton leaves and some water absorption.

#### The PLSR Model Based on R Spectrum and CWT Spectra

To explore the quantitative regression relationship between cotton leaf spectral data and the LKC, a quantitative estimation model was established to realize the quantification of spectral monitoring. Using the R spectrum and CWT spectra composed of characteristic wavelengths screened by CARS and RF as independent variables and the LKC as a dependent variable, PLSR estimation models of the LKC at different growth stages were established (Table 3). The calibration and validation sets perform differently in estimating the effect of the LKC model at different growth stages and decomposition scales. At the budding stage, the model *R*^2^cal and *R*^2^val both were >0.6136 (RF-CWT-7) and 0.2675 (CARS-CWT-4). At the flowering stage, the model *R*^2^cal and *R*^2^val were >0.7311 (RF-CWT-10) and 0.6158 (CARS-CWT-2). In the boll setting period, the model *R*^2^cal and *R*^2^val were both >0.5717 (RF-CWT-3) and 0.5430 (RF-CWT-6).

**Table 3:** CARS and RF algorithms are used to screen the characteristic wavelengths of the wavelet coefficient spectra, the PLSR estimation model of cotton LKC in different growth stages is established, and emphasize the relatively better performances of these the wavelet coefficient spectra in LKC estimation.

**Growth Stage**	**Scales**	**CARS**				**RF**			
		***R* ^2^** **cal**	**RMSE** **cal**	***R* ^2^** **val**	**RMSE** **val**	***R* ^2^** **cal**	**RMSE** **cal**	***R* ^2^** **val**	**RMSE** **val**
Budding stage	R	0.8003	0.3578	0.6613	0.5292	0.7225	0.4217	0.4978	0.6141
	CWT-1	0.8104	0.3485	0.7918	0.3680	0.7537	0.3973	0.7306	0.4421
	CWT-2	0.7568	0.3947	0.5333	0.5737	0.8168	0.3426	0.7157	0.4298
	CWT-3	0.8325	0.3277	0.5471	0.5514	0.7269	0.4183	0.6853	0.4816
	CWT-4	0.6616	0.4657	0.2675	0.7251	0.6257	0.4898	0.3837	0.6761
	CWT-5	0.7274	0.4180	0.5457	0.5823	0.6871	0.4478	0.6176	0.5225
	CWT-6	0.7507	0.3997	0.5647	0.5565	0.6415	0.4793	0.4836	0.6198
	CWT-7	0.7503	0.4000	0.5755	0.5539	0.6136	0.4976	0.5314	0.5943
	CWT-8	0.7545	0.3966	0.5205	0.5722	0.7395	0.4086	0.5549	0.5479
	CWT-9	0.7648	0.3882	0.5200	0.5779	0.6585	0.4678	0.6487	0.4892
	CWT-10	0.7289	0.4168	0.5888	0.5232	0.7333	0.4134	0.5557	0.5549
Flowering stage	R	0.8397	0.1789	0.7530	0.2202	0.8183	0.1905	0.7377	0.2227
	CWT-1	0.8688	0.1619	0.6942	0.2388	0.8017	0.1990	0.7395	0.2190
	CWT-2	0.8585	0.1681	0.6158	0.2627	0.8673	0.1628	0.6757	0.2413
	CWT-3	0.8141	0.1927	0.6598	0.2480	0.8405	0.1785	0.7900	0.1987
	CWT-4	0.8429	0.1771	0.7566	0.2094	0.7571	0.2202	0.6882	0.2444
	CWT-5	0.8007	0.1995	0.7473	0.2187	0.7679	0.2153	0.6279	0.2629
	CWT-6	0.8064	0.1966	0.6772	0.2432	0.7829	0.2082	0.6098	0.2674
	CWT-7	0.8155	0.1919	0.7500	0.2176	0.7453	0.2255	0.5956	0.2708
	CWT-8	0.8074	0.1961	0.7905	0.2070	0.7405	0.2276	0.6960	0.2381
	CWT-9	0.8104	0.1946	0.7710	0.2118	0.7952	0.2022	0.7121	0.2309
	CWT-10	0.8174	0.1910	0.7360	0.2227	0.7311	0.2317	0.6704	0.2477
Boll setting stage	R	0.6487	0.3274	0.6643	0.3405	0.6471	0.3281	0.5476	0.3993
	CWT-1	0.7217	0.2914	0.6441	0.3474	0.8070	0.2426	0.5499	0.4049
	CWT-2	0.7323	0.2858	0.6736	0.3564	0.7493	0.2766	0.6722	0.4169
	CWT-3	0.6212	0.3400	0.6598	0.3229	0.5717	0.3615	0.7481	0.2795
	CWT-4	0.6433	0.3299	0.6869	0.3173	0.6093	0.3453	0.6802	0.3146
	CWT-5	0.5966	0.3508	0.7610	0.2793	0.6182	0.3413	0.6635	0.3301
	CWT-6	0.6380	0.3323	0.7581	0.2820	0.4192	0.4210	0.5430	0.3771
	CWT-7	0.6200	0.3405	0.7254	0.3042	0.5955	0.3513	0.5573	0.3697
	CWT-8	0.6162	0.3422	0.7157	0.3047	0.5794	0.3582	0.7972	0.2734
	CWT-9	0.6310	0.3356	0.8080	0.2508	0.5983	0.3501	0.6917	0.3238
	CWT-10	0.7118	0.2965	0.6867	0.4988	0.6031	0.3480	0.6973	0.3076

**Figure 7:**
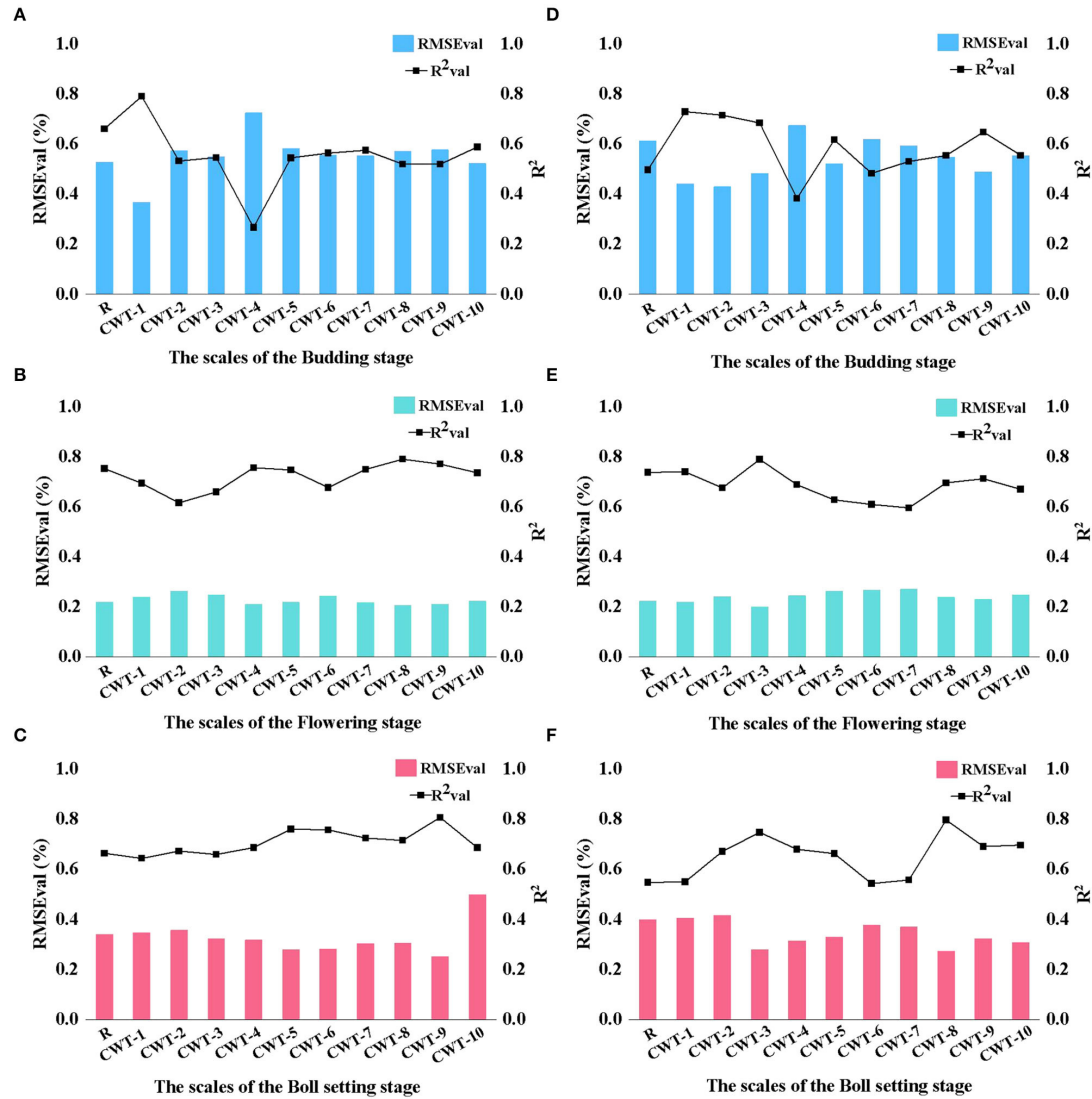
PLSR estimation model of the LKC in cotton based on R and CWT spectra (validation sets). **(A)** CARS-Budding stage. **(B)** CARS-Flowering stage. **(C)** CARS-Boll setting stage. **(D)** RF-Budding stage. **(E)** RF-Flowering stage. **(F)** RF-Boll setting stage.

The all model *R*^2^val and RMSEval results are shown in Figure 7. The results showed that compared with the R spectrum, the CWT spectra could significantly improve the prediction performance of the LKC (Figures 7D–F). The optimal estimation model of the R spectrum at the budding, flowering and boll growth stages was constructed using the characteristic wavelengths selected by the CARS algorithm, indicating that CARS had a better estimation performance than the RF algorithm, this is similar to previous studies (Sun et al., 2021). The *R*^2^val values were 0.6613, 0.753, and 0.6643 and the RMSEval values were 0.5292, 0.2202, and 0.3405, respectively. Using the multi-decomposition scale CWT method, the best decomposition scales of the three growth stages were found to be CWT-1, CWT-3 and CWT-9 spectrum (Table 3). The best estimation models for the LKC were CARS-CWT-1 at the budding stage, RF-CWT-3 at the flowering stage and CARS-CWT-9 at the boll setting stage. The *R*^2^val values were 0.7918, 0.79, and 0.808 and the RMSEval values were 0.368, 0.1987 and 0.2508, respectively (Figures 7A–C). Compared with the single R spectrum model, the improved *R*^2^ values at the three growth stages were 0.13, 0.04, and 0.15, respectively. Higher *R*^2^val values and lower RMSEval values indicate that these models have a good fitting degree and accuracy, and the decomposed CWT spectra can effectively extract weak information, but there are significant differences in the prediction accuracy of the CWT spectra at different decomposition scales, and the screening results of the CARS and RF algorithms also show different model effects.

### Image Feature Analysis

#### Leaf Grayscale Image Database

**Figure 8:**
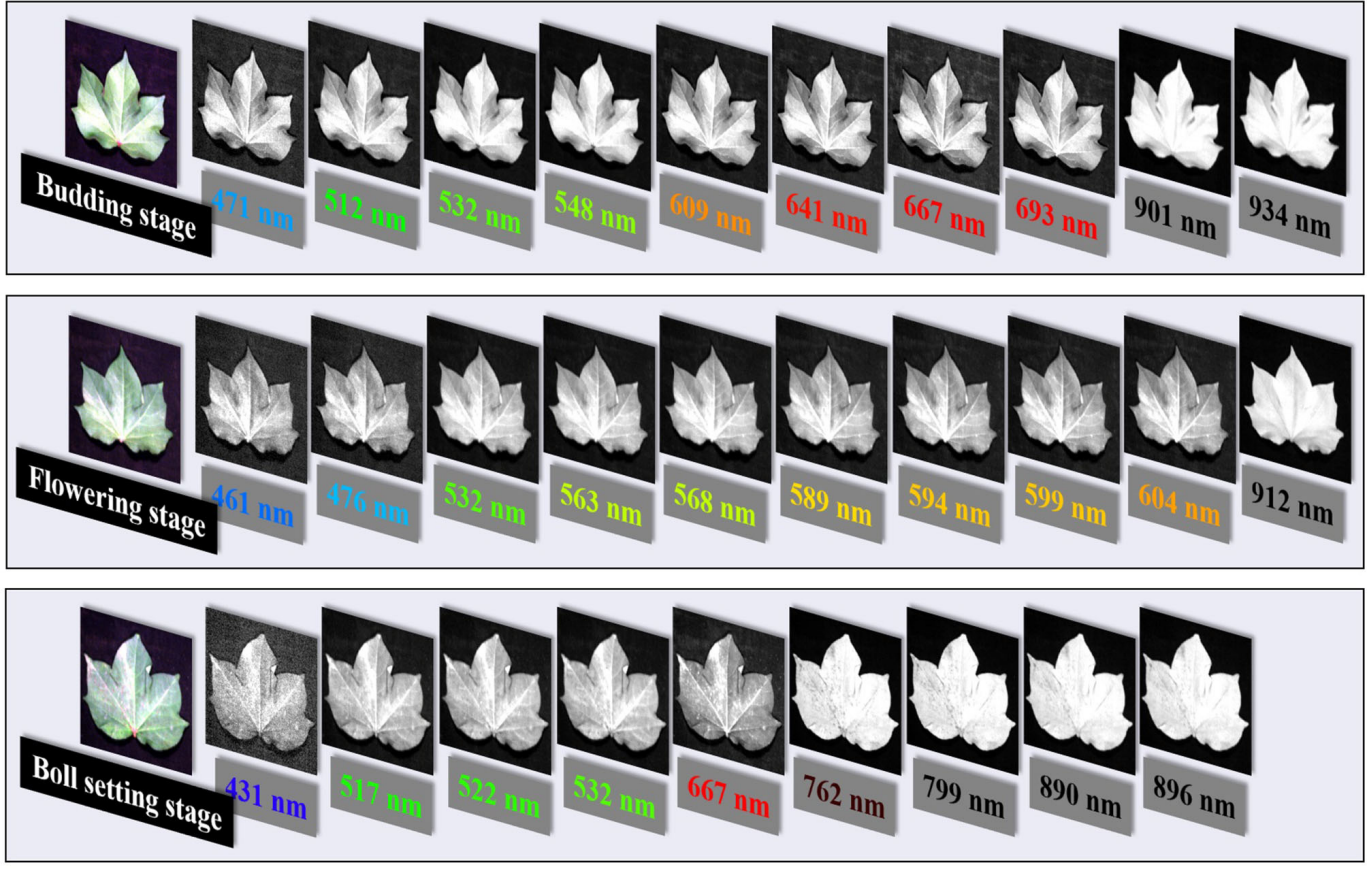
Characteristic wavelength grayscale image databases based on CWT-1, CWT-3, and CWT-9 wavelet coefficients.

To find image features that could optimize the estimation model of the LKC in cotton, gray image databases of leaves at the budding, flowering, and boll setting stages were constructed according to the selected characteristic wavelengths (Figure 8). Among them, the number of effective characteristic wavelengths in the three growth stages are 10, 10, and 9, respectively. Therefore, the total number of grayscale images in budding, flowering, and boll setting stages are 60 × 10, 60 × 10 and 60 × 9, respectively.

#### Image Feature Extraction and Determination

After the mask processing for all images, three color features and 24 texture features of each characteristic wavelength grayscale image were successively calculated. Finally, the color and texture data extracted from the three growth periods were stored in 3 × 30 and 3 × 240 matrices, and a correlation analysis was performed with the LKC, respectively. The results are shown in **Figure 10**, where a square represents a feature.

For color features, the correlation between MEA and VAR was higher than that with SKE at the budding, flowering, and boll setting stages. The highest correlations of color features in the three growth stages were obtained for 532 nm-VAR (*R* = 0.39, *p* < 0.01), which reached extremely significant correlation, 461 nm-MEA (*R* = 0.18, *p* < 0.05) and 522 nm-MEA (*R* = −0.33, *p* < 0.05). The results showed that the overall brightness and color distribution of images were closely related to the LKC. The texture features with a high correlation between the budding, flowering, and boll setting stage were different. The high correlation at the budding stage was mainly the CON of 471, 512, and 641 nm grayscale images in the 90° and 135° directions, and the highest correlation was 641 nm-CON-135° (*R* = −0.48). The results showed that the furrow depth of the cotton leaf surface at the budding stage was negatively correlated with K content. The texture features with high correlation at the flowering stage was mainly the COR of the characteristic wavelength of visible light (e.g., 476 nm) in the direction of 135°, especially 476 nm-COR-135° (*R* = −0.38). During the boll setting stage, highly correlated texture features were concentrated in the CON at the characteristic wavelength of NIR (e.g., 799 nm), and the highest correlation was obtained at 799 nm-VAR (CON) (*R* = −0.44), indicating that the uniformity of the leaf surface texture was significantly negatively correlated with the K at the flowering and boll setting stages.

**Figure 9:**
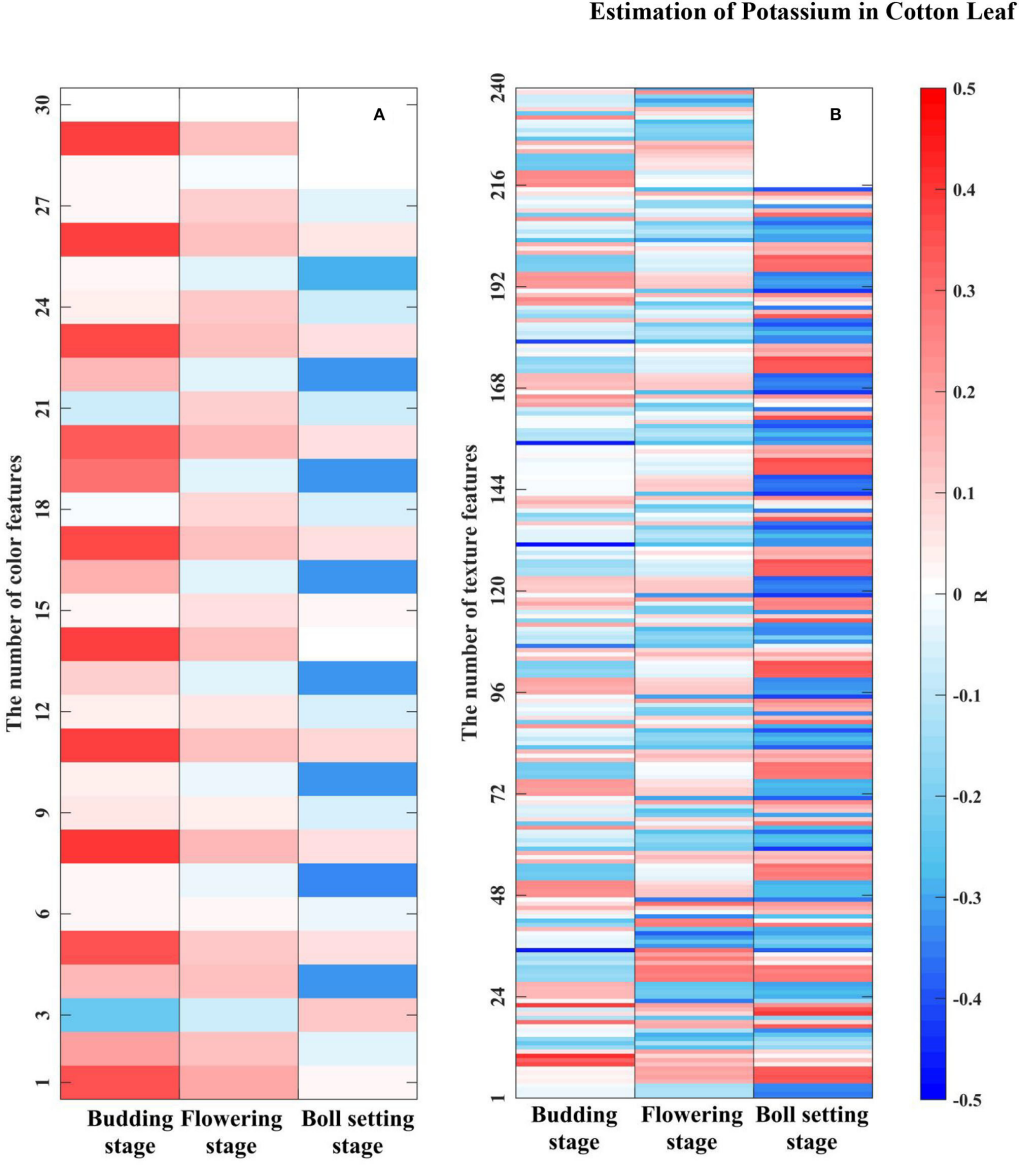
Correlation analysis of the LKC with grayscale image features. **(A)** Color features. There were 10 × 3, 10 × 3 and 9 × 3 color features in three growth stages, respectively. **(B)** Texture features. There were 10 × 24, 10 × 24, and 9 × 24 texture features in three growth stages, respectively.

As can be seen from Figure 9, the color and texture feature dimensions of each growth period have higher dimensions and contain a large amount of invalid information. Therefore, we choose the CARS algorithm with better performance and combined correlation analysis to determine the effective image features. The screening results are shown in Table 4.

**Table 4:** Grayscale image feature extraction results.

**Growth Stage**	**Image**	**Number**	**Combined Feature Name**
	**Feature**		
Budding	Color	1	548 nm-MEA
stage			471 nm-CON-90°, 471 nm-CON-0°,
	Texture	6	934 nm-ENT-45°, 471 nm-COR-135°, 641 nm-CON-135°, 512 nm-ENT-135°
Flowering	Color	4	461 nm-MEA, 532 nm-SKE, 589
stage			nm-MEA, 599 nm-SKE
			532 nm-ENE-135°, 568 nm-COR-135°,
	Texture	8	912 nm-VAR (COR), 594 nm-MEA (CON), 532 nm-MRA (CON), 599 nm-ENT-0°, 604 nm-ENE-90°, 461 nm-MEA (COR)
Boll setting	Color	2	667 nm-VAR, 532 nm-VAR
stage	Texture	3	799 nm-ENE-135°, 522 nm-ENT-90°, 890 nm-ENT-135°

### Model Establishment and Validation

#### Estimation Model of the LKC Based on Combination Feature

Table 5 shows the results of the PLSR estimation model of the LKC in cotton at different growth stages was created based on the combined characteristics of “CWT spectra + image.” Compared with the R spectrum and CWT spectra models, the fusion of image features can improve the accuracy of the K estimation model at the three growth stages, but the performance of models constructed based on CWT spectra fusion with either “color” or “texture” or with “color + texture” features is different. Among them, the best estimation model was “CWT-1 + texture” for the budding stage, “CWT-3 + color” for the flowering stage and “CWT-9 + texture” for the boll setting stage. Based on the “spectra + image” combination, the best estimation models of the LKC in cotton at the budding, flowering, and boll setting stages had 16, 14, and 12 features, respectively. Moreover, texture features contribute more to the model performance improvement than color features do. These results provide a reference for rapid and non-destructive monitoring of the K.

**Table 5:** PLSR estimation models of the LKC with different combinations of features.

**Growth Stage**	**Method**	**Feature Combination**	**Calibration sets**		**Validation sets**	
			***R*^2^cal**	**RMSEcal**	***R*^2^val**	**RMSEval**
Budding stage	CARS	R spectrum	0.8003	0.3578	0.6613	0.5292
		CWT-1	0.8104	0.3485	0.7918	0.3680
		CWT-1 + color	0.8124	0.3467	0.8127	0.3488
		CWT-1 + texture	0.8017	0.3564	0.8652	0.3009
		CWT-1+ color + texture	0.8344	0.3258	0.7980	0.3602
Flowering stage	CARS	R spectrum	0.8397	0.1789	0.7530	0.2202
	RF	CWT-3	0.8405	0.1785	0.7900	0.1987
	CARS	CWT-3 + color	0.8530	0.1714	0.8261	0.1821
		CWT-3 + texture	0.8400	0.1788	0.8012	0.1952
		CWT-3 + color + texture	0.8621	0.1660	0.7960	0.2007
Boll setting stage		R spectrum	0.6487	0.3274	0.6643	0.3405
		CWT-9	0.6310	0.3356	0.8080	0.2508
	CARS	CWT-9 + color	0.6333	0.3345	0.8111	0.2463
		CWT-9 + texture	0.6948	0.3052	0.8952	0.2019
		CWT-9 + color + texture	0.7603	0.2704	0.8272	0.2450

#### Comparison of Model Between Single Spectrum and “Spectrum + Image” Feature

**Figure 10:**
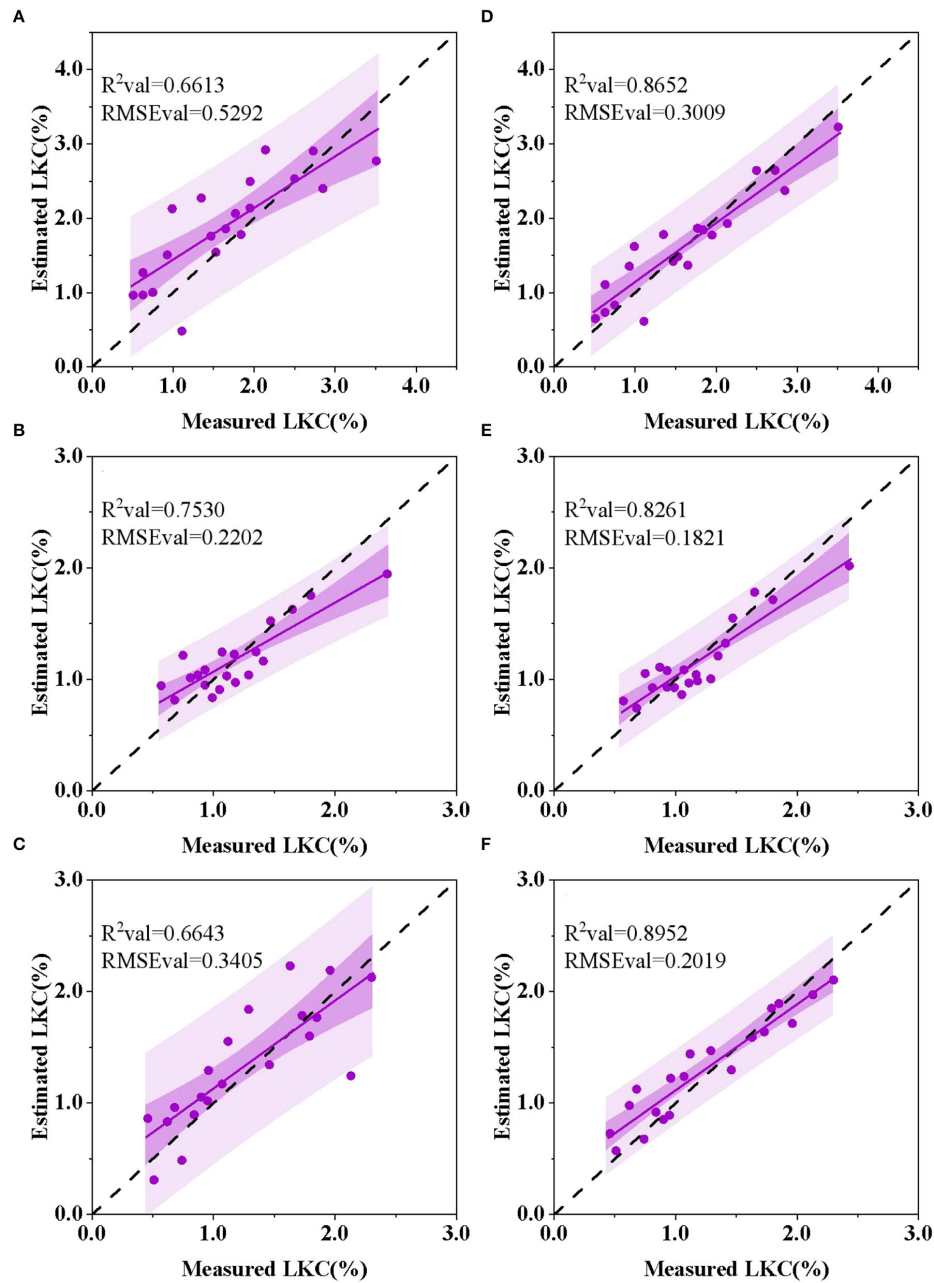
Optimal PLSR estimation model of the LKC in cotton at different growth stages. **(A)** Budding stage, R spectrum. **(B)** Flowering stage, R spectrum. **(C)** Boll setting stage, R spectrum. **(D)** Budding stage, CWT-1 + texture. **(E)** Flowering stage, CWT-3 + color. **(F)** Boll setting stage, CWT-9 + texture.

Figure 10 shows the best inversion model of the LKC in cotton at three growth stages. The optimal LKC estimation model constructed based on the characteristic wavelengths of R spectra at budding, flowering, and boll setting stages had *R*^2^val s of 0.6613, 0.7530, and 0.6643; and RMSEvals of 0.5292, 0.2202, and 0.3405, respectively (Figures 10A–C). Based on the “CWT spectra + image” feature, the accuracy *R*^2^val values of the best LKC estimation model for the three growth stages had *R*^2^val s of 0.8652, 0.8261, and 0.8952 and the RMSEval of 0.3009, 0.1821, and 0.2019, respectively (Figures 10D,F). Compared with the single R spectrum, the model accuracy *R*^2^ increased by 0.2, 0.08, and 0.23, respectively, indicating the feasibility of estimating the LKC in cotton based on CWT analysis and image feature fusion and achieving high-precision and rapid monitoring. The best estimation of the LKC in cotton was at the boll setting stage. Furthermore, because the fusion image features at the flowering stage contributed little to the improvement of the model accuracy, it indicated that the quantitative inversion of the K in the growth stage could be satisfied only by R spectral data, and the model had high stability.

## Discussion

### Selection of Characteristic Wavelength

In our study, there was a high correlation between CWT spectra with the LKC at the three key growth stages of cotton. However, it should be noted that LKC in cotton is not highly correlated with wavelet coefficients in the whole band of 400–950 nm but only in some important spectral regions (Figure 5). Therefore, to reduce collinearity between spectral data dimensions and adjacent wavelengths, screening several effective wavelengths containing maximum spectral information plays an important role in reducing model complexity and improving estimation ability (Lu et al., 2019b; Ruffing et al., 2021). In this study, characteristic wavelengths of the R spectrum and CWT spectra at three growth periods were screened based on the CARS and RF algorithms (Table 3). In general, the characteristic wavelengths were mainly concentrated in the visible and NIR regions in the whole spectrum. The research found that the characteristic wavelengths of the LKC of six degraded vegetation types in the green, red and NIR regions (Peng et al., 2020). Studies have shown that the K deficiency in leaves has a significant impact on the content of photosynthetic pigments (e.g., chlorophyll, carotenoid, and lutein), and the cell structure of leaves (e.g., leaf area, leaf thickness, and cell space) (Curran, 1989; Hu et al., 2020a,b), which is a key factor affecting the light absorption and utilization of plant leaves, leading to the change in reflectance (Peuelas and Filella, 1998). When crops are subjected to the K stress, the spectral reflectance of the visible and near-infrared regions increases, while chlorophyll concentration decreases (Zhao et al., 2001). At the same time, the chlorophyll ultrastructure is significantly damaged, leaf thickness increases, palisade tissue and parenchyma cells contract, and local rupture occurs (Lu et al., 2019a). In conclusion, the K deficiency symptoms can significantly affect the absorption and reflection of light by cotton leaves, change the path of light reflection and refraction and produce different spectral reflectance curves. In addition, studies have shown that the spectral reflectance of the SWIR (1,300–2,000 nm) band of rice was sensitive to K level and significantly correlated with the LKC in rice (Lu et al., 2019a). Pimstein et al. (2011) pointed out that the SWIR (1,450 nm) reflectance was significantly correlated with LKC in wheat. Sibanda et al. (2015) also showed that the SWIR spectroscopy can be used to determine *K* value defense on steppe.

### Estimation of the LKC Based on CWT Algorithm

The CWT can decompose hyperspectral data in the time domain and frequency domain simultaneously and estimate the physiological and biochemical components of plants by looking for the best signals at different decomposition scales. The estimation model of the LKC in cotton constructed in this study using CTW spectral data has good prediction accuracy. Based on spectral data, the accuracy *R*^2^ value of the best CWT spectral models constructed for the three growth stages was 0.13, 0.04, and 0.15 higher, than that of the R spectral data model (Figure 7). Since CWT can further continuously decompose spectral data, the decomposed wavelet coefficients can correspond to the R spectrum so as to extract subtle signals in spectral data more effectively and improve spectral monitoring accuracy (Li et al., 2019). However, it should be noted that when using the CWT method, the mother wavelet function should be selected first, rather than the commonly used mother wavelet function (Sun et al., 2021; Zhao et al., 2021). A large number of studies have shown that spectral data transformed by CWT have achieved a high accuracy in the inversion of crop nutrients, chlorophyll, and agronomic traits that is superior to models obtained by traditional conversion methods (Yue et al., 2020; Lin et al., 2021; Zhao et al., 2021). Therefore, when crops are under nutrient stress, CWT can effectively mine more complete spectral information, which has great potential in feature selection, noise elimination and weak information extraction.

### Estimation Model of the LKC in Cotton

The single feature extracted from the hyperspectral image has limited abilities to estimate nutrient content. Our comparison of the model performance evaluation resulting from different feature combinations (Table 5) showed that the estimation of the LKC in cotton based on “CWT spectra + image” features had high accuracy and stability at the three key growth stages, but there were differences in the modeling results of different feature combinations. Contrary to our hypothesis, it was not the case that more feature information led to better model performance. In this study, the estimation model constructed based on the features of “CWT spectra + color + texture” did not show significantly improved accuracy. Instead, the performance of models based on “CWT spectra + color” or “CWT spectra + texture” features is improved. It indicates that when constructing the model based on the feature information of “CWT spectra + color + texture,” some invalid information is added, which is interference for improving the accuracy and stability of the prediction model (Li et al., 2019). It should be noted that although the combination feature of the best estimation model of the LKC at the flowering stage is “cwt-3 + color,” and the model accuracy *R*^2^val and RMSEval are 0.8261 and 0.1821, the absolute values of the difference between *R*^2^val *R*^2^val and RMSEval based on the “CWT-3 + color” and “CWT-3 + texture” models are 0.0249 and 0.0131 (Table 5). This slight difference may be influenced by the feature parameter selection algorithm, as can be seen from Table 4, the number of color features selected by CARS during the flowering stage is relatively a little more than in other growth periods. On the other hand, statistically speaking, the difference between the two models is negligible. Therefore, it cannot be fully stated that the combined characteristics of the best estimation model for the LKC at the flowering stage is “CWT-3 + color.” Moreover, this study aimed at extracting image features of grayscale images with feature wavelengths, and different feature selection methods may obtain different feature wavelengths (Sun et al., 2021), which will lead to extracting different image feature values. Therefore, future studies will further explore the relationship between the selection of feature wavelengths and image features.

Compared with texture, color features did not contribute significantly to the estimation of the LKC. This is similar to the results of the Jamil et al. (2015) study, which showed that in the taxonomic identification of 455 Chinese herbal medicines, single texture features were superior to color or shape features, with a recognition rate of 92%. In our study, the reason for this discrepancy may be that the image we studied was a single-band grayscale image that contained different information about color and texture characteristics. For color features, we use low-dimensional color moments composed of mean, variance, and skewness to represent the color characteristics of single-band grayscale images, and because the number of channels is 1, the number of color features obtained by calculation is relatively small. In addition, for the color features we extracted, it can also be considered as another expression of spectral information, because each grayscale image has a corresponding wavelength, and perhaps there is interference information between them, reducing the additional effect of color features. However, further investigation needs to be done to positively confirmed the claim. For texture features, we used the GLCM algorithm to extract 240, 240, and 216 high-dimensional texture features from three reproductive periods, including 4 texture features in 4 directions, and its advantage may be that a larger number of feature parameters are conducive to the selection of sensitive parameters. Further, images of different wavelengths of grayscale can clearly show the veins and mesophyll parts of cotton leaves and their degree of brightness and shade (Figure 8), while texture is another feature that can be used in plant identification to describe the vein structure or leaf's surface, and it is considered as an additional feature to better describe properties of the leaves (Jamil et al., 2015). These reasons explain, as far as possible, why when we build cotton LKC estimation models, the texture features are better than the color features, and the mechanism of the relationship between LKC and texture features needs further study. Therefore, near-range hyperspectral images with high spatial and hyperspectral resolution can provide more details (Pandey et al., 2017), the model based on “CWT spectra + image” features provide a potential method for estimating the LKC in cotton.

Previous studies mainly focused on using hyperspectral data (Das et al., 2020; Furlanetto et al., 2021) or RGB images (Ghosal et al., 2018; Sun et al., 2018) for analytical modeling of the estimation of the LKC in crop. Ge et al. (2019) constructed a low-cost, non-destructive, and high-throughput maize multiphysiological parameter (including K) estimation model based on the full spectrum band (VIS-NIR-SWIR) through PLSR and SVM methods. The results for K nutrients show that the modeling results of PLSR are similar to those of SVM, and the and the model accuracy *R*^2^val is 0.586 and 0.543, respectively. The performance of the model largely depends on the sensitivity of input parameters. Compared with their research, we established the model based on the characteristic wavelength sensitive to the LKC in cotton, rather than the full wavelength, but we do not have the SWIR region, which is a deficiency. Although our study used a PLSR model to estimate the LKC with good robustness, the analysis of hyperspectral image data for large samples needs to be further explored, especially as the continuous optimization of deep learning algorithms may be beneficial to LKC estimation (Mahajan et al., 2021; Mertens et al., 2021). Das et al. (2020) studied the content changes of eight nutrient elements (K, Na, Ca, Mg, Fe, Mn, Zn, and Cu) in rice leaves under salt stress and constructed different coupled machine learning models. The results showed that the most accurate estimation of the LKC based on the PLSR-ELNET model (*r* = 0.928). Liu et al. (2020a) proposed a novel ensemble-modeling framework to transform the rape canopy reflectance data of the selected bands into more distinguishable probability features and identify the N, P, and K deficiency levels using the probabilities. The overall accuracy of nutritional deficiency analysis of this framework is 80.76%, it shows a competitive advantage in severe and moderate potassium deficiency. In this regard, we will study more predictive model algorithms in future work to provide a reference for rapid and non-destructive monitoring of the LKC in cotton.

## Conclusions

The CWT method and PLSR model were used to estimate the LKC in cotton, which had high spectral prediction accuracy and feasibility. The CARS and RF algorithms combined with the PLSR model were used to determine the optimal decomposition scales of CWT at three growth stages, which were CWT-1, CWT-3, and CWT-9 spectra. Also, the CARS is better than RF in Characteristics selection. Compared with the single R spectrum model, the *R*^2^ values were improved by 0.13, 0.04, and 0.15, respectively.

Compared with the best estimation model of the R spectrum and CWT spectra, the PLSR model accuracy was improved after the fusion of image features at the three growth stages. The best feature combination of estimation models was “CWT-1 + texture” at the budding stage, “CWT-3 + color” at the flowering stage and “CWT-9 + texture” at the boll setting stage. Compared with the single R spectrum model, the accuracy *R*^2^ values increased by 0.2, 0.08, and 0.24, respectively.

Using characteristic wavelength to fuse image information can optimize the performance of the LKC estimation model and improve the stability and accuracy of the model. Based on the combination of “CWT spectra + image,” the best growth stage for assessing LKC in cotton was the boll setting stage, with the feature combination of “CWT-9 spectra + texture,” and its *R*^2^val and RMSEval values were 0.90 and 0.20. However, the optimal growth stage for estimating LKC only by R spectral estimation is the flowering stage. The model did not show significantly improved prediction accuracy and had high stability after integrating image features, indicating that the quantitative estimation of the LKC based on spectral data can be satisfied in this growth stage.

## Data Availability Statement

The raw data supporting the conclusions of this article will be made available by the authors, without undue reservation.

## Author Contributions

QY conceived the study, analyzed the data, and wrote the manuscript. CS finished the laboratory experiment. XC and LM trained the algorithms. ZZ and XL proofread and made comments on the manuscript. All authors discussed in the review process and contributed to the article and approved the submitted version.

## Funding

This study was supported by the National Natural Science Foundation of China (42061058) and the Corps' key areas of research projects (2020AB005).

## Conflict of Interest

The authors declare that the research was conducted in the absence of any commercial or financial relationships that could be construed as a potential conflict of interest.

## Publisher's Note

All claims expressed in this article are solely those of the authors and do not necessarily represent those of their affiliated organizations, or those of the publisher, the editors and the reviewers. Any product that may be evaluated in this article, or claim that may be made by its manufacturer, is not guaranteed or endorsed by the publisher.
